# Supplementary material for: A set of multi-entry identification keys to African frugivorous flies (Diptera, Tephritidae)
Source: Zookeys. 2014 Jul 24;(428):97–108. doi: 10.3897/zookeys.428.7366 (PMC4143993; doi:10.3897/zookeys.428.7366)
Supplement: Supplementary material 6 — Key to Ceratitis [file zookeys-428-097-s006.zip › SF6_ZooKeys_key to Ceratitis/key/SF6_ZooKeys_key to Ceratitis/Media/Html/Ceratitis fasciventris.htm]

Ceratitis (Pterandrus) fasciventris (Bezzi)


***Ceratitis*** **(*Pterandrus*) *fasciventris*
(Bezzi)**

*Pterandrus
rosa* var.
*fasciventris* Bezzi, 1920: 228.

Body
length: 4.47 (3.95-5.15) mm; wing length: 4.96 (4.45-5.75) mm.

Male

Head: Antenna yellowish orange. First flagellomere 2-3 times
as long as pedicel. Arista with short to moderately long rays; ventral rays
shorter and sparser than dorsal rays, especially basally. Frons yellow; with
short scattered setulae distinctly darker than frons. Frontal setae well
developed. Face yellowish white. Genal seta and setulae dark, well developed.

Thorax: Postpronotal
lobe yellowish white, without spot. Mesonotum: ground color dark gray,
sometimes with orange tinge; with streaks and darker markings but without
distinct spots, except prescutellar white separate markings, usually with paler
gray area in between, occasionally merged. Scapular setae dark. Scutellum
yellowish white, basally usually without dark spots, rarely with two separate
spots; apically with three separate dark spots, extending to basal half,
sometimes to basal 0.33. Anepisternum on ventral half yellowish brown; setulae
pale.

Legs:
Yellow except where otherwise noted; setation typical for subgenus, mixed pale
and dark. Foreleg: at most slightly darker yellow anteriorly; femur without
bushy feathering posteriorly, only row of dispersed, long and usually black
setulae posteriorly, setulae shorter and pale posterodorsally and
posteroventrally; ventral setae black. Midleg: femur with dispersed pale
setulae at base ventrally; tibia not broadened; anteriorly with inconspicuous
silvery shine on distal half when viewed from certain angle; ventrally and
dorsally with dark feathering along distal half. Hindleg: femur at apical 0.25
with longer setulae dorsally and ventrally.

Wing:
bands brown or yellowish brown. Interruption between marginal and discal bands
near vein R_1_ clear and complete; cubital band free; medial band
absent; crossvein R-M opposite middle of discal cell, sometimes just proximal
to middle. Apex of vein R_1_ distal to level of crossvein R-M.
Crossvein DM-Cu oblique anterobasally.

Abdomen:
Mostly yellow. Tergites 2 and 4 with pale gray band on posterior half, anterior
margin sometimes with narrowly brownish colored. Tergite 3 with distinct
brownish black transverse band along� posterior half; rarely more complete
brown. Tergite 5 with basal half brownish, sometimes divided medially into two
spots, or only narrowly brownish along anterior margin. Male epandrium in
lateral view with posterior lobe of lateral surstylus short and straight,
anterior lobe well pronounced.

Female

As
male except for the following characters: Anepisternum on ventral half brown or
yellowish brown. Crossvein DM-Cu variable. Legs without feathering; forefemur
with posterodorsal row usually partly dark; forefemur posteroventrally with pale
pilosity. Oviscape shorter than preabdomen. Aculeus at most six times longer
than wide; tip with distinct apical indentation and lateral margin slightly
sinuous.

(Description
after De Meyer & Freidberg, 2006)
